# Chloroquine, an Endocytosis Blocking Agent, Inhibits Zika Virus Infection in Different Cell Models

**DOI:** 10.3390/v8120322

**Published:** 2016-11-29

**Authors:** Rodrigo Delvecchio, Luiza M. Higa, Paula Pezzuto, Ana Luiza Valadão, Patrícia P. Garcez, Fábio L. Monteiro, Erick C. Loiola, André A. Dias, Fábio J. M. Silva, Matthew T. Aliota, Elizabeth A. Caine, Jorge E. Osorio, Maria Bellio, David H. O’Connor, Stevens Rehen, Renato Santana de Aguiar, Andrea Savarino, Loraine Campanati, Amilcar Tanuri

**Affiliations:** 1 Department of Genetics, Institute of Biology, Federal University of Rio de Janeiro, Rio de Janeiro 21941-902, Brazil; digodelvecchio@gmail.com (R.D.); luizahiga@gmail.com (L.M.H.); paulapezzuto81@gmail.com (P.P.); analuvaladao@gmail.com (A.L.V.); fabiolimonte@gmail.com (F.L.M.); santanarnt@gmail.com (R.S.d.A.); 2 Institute of Biomedical Sciences, Federal University of Rio de Janeiro, Rio de Janeiro 21941-902, Brazil; ppgarcez@gmail.com (P.P.G.); fabiojms@icb.ufrj.br (F.J.M.S.); srehen@lance-ufrj.org (S.R.); 3 D’Or Institute for Research and Education (IDOR), Rio de Janeiro 22281-100, Brazil; erickloiola@lance-ufrj.org; 4 Department of Immunology, Federal University of Rio de Janeiro, Rio de Janeiro 21941-902, Brazil; aadias2005@yahoo.com.br (A.A.D.); mariabellioufrj@gmail.com (M.B.); 5 Department of Pathobiological Sciences, University of Wisconsin-Madison, Madison, WI 53706, USA; mtaliota@wisc.edu (M.T.A.); eacaine@wisc.edu (E.A.C.); jorge.osorio@wisc.edu (J.E.O.); 6 Department of Pathology and Laboratory Medicine, University of Wisconsin-Madison, Madison, WI 53706, USA; dhoconno@wisc.edu; 7 Istituto Superiore di Sanità, Deptartment of Infectious Diseases, 299 Viale Regina Elena, 00161 Rome, Italy; ansavari@yahoo.com

**Keywords:** Zika virus, chloroquine, antiviral, microcephaly, neural stem cell

## Abstract

Zika virus (ZIKV) infection in utero might lead to microcephaly and other congenital defects. Since no specific therapy is available thus far, there is an urgent need for the discovery of agents capable of inhibiting its viral replication and deleterious effects. Chloroquine is widely used as an antimalarial drug, anti-inflammatory agent, and it also shows antiviral activity against several viruses. Here we show that chloroquine exhibits antiviral activity against ZIKV in Vero cells, human brain microvascular endothelial cells, human neural stem cells, and mouse neurospheres. We demonstrate that chloroquine reduces the number of ZIKV-infected cells in vitro, and inhibits virus production and cell death promoted by ZIKV infection without cytotoxic effects. In addition, chloroquine treatment partially reveres morphological changes induced by ZIKV infection in mouse neurospheres.

## 1. Introduction

Zika virus (ZIKV) is an arthropod-borne virus, transmitted by *Aedes* mosquitoes, that belongs to the *Flavivirus* genus, which also includes other pathogens such as West Nile virus (WNV), yellow fever virus (YFV), Japanese encephalitis virus (JEV), and dengue virus (DENV). Zika virus was first isolated from a sentinel monkey in the Zika forest in Uganda in 1947 [1]. Since then, ZIKV has been isolated from humans and mosquitoes throughout Africa and Southeast Asian countries. Phylogenetic analysis of the nonstructural protein 5 encoding region has disclosed three ZIKV lineages: East African, West African, and Asian [2]. Sequences from Uganda and Senegal belong to the East African cluster, while sequences from Nigeria and also some sequences from Senegal are clustered on the West African lineage [3]. Most of the viral sequences recovered from recent outbreaks, from 2007 until now, belong to the Asian lineage, such as strains isolated in American, South Asian, and Pacific countries. In utero exposure to Asian lineage ZIKV might lead to microcephaly and other developmental malformations including calcifications, arthrogryposis, ventriculomegaly, lissencephaly, cerebellar atrophy, and ocular abnormalities [4,5,6,7,8]. Although all ZIKV lineages can infect humans, these severe manifestations reported after in utero infection have only been associated with Asian lineages, including Brazilian isolates [6,9]. 

Asian ZIKV strains were detected in the brain and amniotic fluid of newborns and stillborns with microcephaly [4,5,6,8,9] and the African MR766 strain was shown to kill human neuroprogenitor cells in vitro as well as decrease the growth rate of brain organoids [10,11].

Symptoms of infections with the Asian ZIKV include low-grade fever, headache, rash, conjunctivitis, arthritis, and myalgia [4,12]. Mild symptoms such as headache and low-grade fever were reported by a human volunteer during infection with the African ZIKV strain [13]. However, in rare instances, infection with the Asian ZIKV is associated with cases of Guillain–Barré syndrome [14] and meningoencephalitis [15]. Currently, there is no vaccine or specific therapeutic approaches to prevent or treat ZIKV infections. With the alarming increase in the number of countries affected and the potential for viral spread through global travel and sexual transmission [16,17], there is an urgent need to find a treatment capable of lessening the effects of the disease and inhibiting further transmission.

Chloroquine, a 4-aminoquinoline, is a weak base that is rapidly imported into acidic vesicles, increasing their pH [18]. It is approved by the Food and Drug Administration (FDA) to treat malaria and has long been prophylactically prescribed to pregnant women at risk of exposure to *Plasmodium* parasites [19]. Chloroquine, through the inhibition of pH-dependent steps of viral replication, restricts human immunodeficiency virus (HIV) [20], influenza virus [21], DENV [22], JEV [23], and WNV infection [24]. Here we investigated the antiviral effects of chloroquine on both Asian (using a Brazilian isolate) and African ZIKV infections in different cell types. 

## 2. Materials and Methods 

### 2.1. Cell Culture

Vero cells (ATCC, Manassas, VA, USA) are derived from the kidney of African green monkey and were grown in DMEM High Glucose (GIBCO, Thermo Fisher Scientific, Waltham, MA, USA) supplemented with 5% fetal bovine serum (FBS) (GIBCO). Human brain microvascular endothelial cells (hBMEC) were a kind gift from Dr. Julio Scharfstein (Federal University of Rio de Janeiro, Rio de Janeiro, Brazil), and hBMEC isolation was performed as previously described [25]. These cells were cultured in DMEM High Glucose supplemented with 20% FBS. The C6/36 cell line is derived from *Aedes albopictus*. C6/36 cells (ATCC, Manassas, VA, USA) were grown in Leibovitz L-15 medium (GIBCO) supplemented with 2.95 g/L tryptose phosphate broth (Sigma Aldrich, Boston, MA, USA), 2 mM glutamine (GIBCO), 0.075% sodium bicarbonate (GIBCO), 1X non-essential amino acids (GIBCO) and 5% FBS. Neural stem cells (NSCs) were derived from human induced pluripotent stem cells (iPSCs). iPSCs were provided by the Biobank of iPSCs of the Brazilian Ministry of Health (CONEP B-027 # 25000.111598/2014-04). According to the supplier, fibroblasts were reprogrammed using the protocol developed by Paulsen et al. [26], and transduced with the CytoTune-iPS Sendai kit (Thermo Fisher Scientific, Waltham, MA, USA). iPSCs presented a normal karyotype and the expression of pluripotency markers. These cells were cultured with E8 culture media (GIBCO) on a Matrigel (BD Biosciences, San Jose, CA, USA) coated surface. iPSC colonies were manually passaged every 5–7 days until they reached 70%–80% confluence and were maintained at 37 °C in humidified air with 5% CO_2_. To produce NSCs, human iPSCs were exposed to the serum-free neural induction medium (GIBCO), containing Neurobasal medium (GIBCO) and the pluripotent stem cell neural induction supplement (GIBCO), according to the manufacturer’s protocol [27]. Briefly, the medium was changed every other day until day 7, when initial NSCs are split and grown on neural expansion medium (Advanced DMEM/F12 and neurobasal medium (1:1) with neural induction supplement; GIBCO). NSCs were used after four passages in neural expansion media. Mouse central nervous system (CNS) cells were harvested from Swiss mouse embryos at embryo day 14 (E14) and grown for 72 h as free floating neurospheres in neurobasal culture media (GIBCO) supplemented with 1X B27 (GIBCO).

### 2.2. Compounds

Chloroquine diphosphate was kindly supplied by FarManguinhos (Fiocruz, Rio de Janeiro, Brazil). The lyophilized powder was diluted in double distilled water to 20 mM. The chloroquine solution was filtered through a 0.22 μm membrane and stored at −80 °C.

### 2.3. Viruses

ZIKV strain MR 766 (Uganda/Africa, accession no.: NC012532.1, kindly provided by Dr. Davis Ferreira, Federal University of Rio de Janeiro, Rio de Janeiro, Brazil) was isolated from a rhesus monkey and injected intracerebrally on Swiss mice for several passages [1] and ZIKV BR (Recife/Brazil, ZIKV PE/243, accession no: KX197192.1, kindly provided by Dr. Marli Tenório Cordeiro, Centro de Pesquisas Aggeu Magalhães, Recife, Brazil) was isolated from a patient presenting classical symptoms of ZIKV infection [28]. These viruses were propagated in Vero and C6/36 cells, respectively. Briefly, the cells were infected with ZIKV at a multiplicity of infection (MOI) of 0.01 and incubated at 37 °C. After 1 h, the inoculum was removed and replaced with DMEM high-glucose (Vero) and Leibovitz L-15 (C6/36) growth media supplemented with 2% FBS. After 4 to 6 days, the conditioned medium was harvested, centrifuged at 300× *g*, and sterile-filtered to remove cells and cellular debris. Virus stocks were stored at −80 °C.

### 2.4. Plaque Assay

Virus titers were determined by plaque assay performed on Vero cells. Virus stocks or samples were serially diluted and adsorbed to confluent monolayers. After 1 h, the inoculum was removed and cells were overlaid with semisolid medium constituted of alpha-MEM (GIBCO) containing 1% carboxymethyl cellulose (Sigma Aldrich) and 1% FBS (GIBCO). Cells were further incubated for 5 days when cells were fixed in 4% formaldehyde. Cells were stained with 1% crystal violet in 20% ethanol for plaque visualization. Titers were expressed as plaque forming units (PFU) per milliliter.

### 2.5. Quantification of ZIKV-Infected Cells by Flow Cytometry

Vero cells, hBMEC, and NSC were infected with ZIKV MR 766 or ZIKV BR strain at an MOI of 2. After 1 h, the inoculum was removed and medium containing chloroquine (6.25 to 50 μM) was added to the cells. After 4 to 5 days, cells were fixed with 4% paraformaldehyde (Sigma Aldrich) in phosphate buffered saline (PBS) for 15 min at room temperature and washed with PBS. Cells were permeabilized with 0.1% Triton X-100 (Sigma Aldrich) in PBS, washed with PBS, and blocked with PBS with 5% FBS. Cells were incubated with 4G2, a pan-flavivirus antibody raised against the ZIKV envelope E protein produced in 4G2-4-15 hybridoma (ATCC), diluted 1:5 in PBS with 5% FBS. Cells were labeled with donkey anti-mouse Alexa Fluor 488 antibody (Thermo Scientific, Waltham, MA, USA) diluted 1:1000 in PBS with 5% FBS, and were analyzed by flow cytometry in a BD Accuri C6 (Becton, Dickinson and Company, Franklin Lakes, NJ, USA) for ZIKV infection. 

### 2.6. Immunofluorescence Assay

Vero, hBMEC, and NSC were seeded on black 96-well plates with clear bottoms and infected with ZIKV MR 766 or ZIKV BR strain at an MOI of 2 for 1 h. Neurospheres were seeded on coverslips and infected with ZIKV MR766 with 2.5 × 10^5^ PFU After infection, the viral inoculum was removed and cells were incubated with medium containing chloroquine (1.56 to 50 μM) for 4 to 5 days, depending on cell type. Cells and neurospheres were fixed with 4% paraformaldehyde in PBS for 20 min at room temperature. The fixative was removed and the samples were washed three times with PBS. Blocking of unspecific binding of the antibody and permeabilization were performed with PBS supplemented with 3% bovine serum albumin (BSA, Sigma Aldrich) and 0.1% Triton X-100 for 40 min at room temperature. Incubation with anti-Map2 antibody was performed following the manufacturer’s instructions (ABCAM, Cambridge, Cambridgeshire, UK, #32454; 1:1000) and the 4G2 antibody was diluted 1:4 in PBS with 3% BSA and incubated with cells for 1 h. After washing three times with PBS, cells were incubated with secondary antibodies coupled to Alexa fluorochromes (Thermo Fisher Scientific) for 40 min and washed five times. Coverslips with neurospheres were mounted with Prolong Gold mounting medium (Thermo Fisher Scientific). Samples were imaged using either Leica SP5 or Leica SPE (Leica Biosystems, Wetzlar, Hesse, DE) confocal microscopes and a Nikon TE300 (Tokyo, Japan) inverted microscope coupled to a Leica DFC310FX camera (Leica Biosystems, Wetzlar, Germany). 

### 2.7. Virus Infection Inhibition Through Cell Viability Assay

Vero, hBMEC, and NSC were exposed to ZIKV MR 766 at an MOI of 2. After 1 h, inoculum was removed and chloroquine-containing medium (6.25 to 50 μM) was added to the cells. Five days post-infection, 20 μL of CellTiter Blue reagent (Promega, Madison, WI, USA) was added in each well, incubated for 12–16 h, and fluorescence was measured (560/590 nm), except for NSCp14 cells when CellTiter Blue was added at three days post-infection. Mean fluorescence intensity (MFI) and standard deviation were displayed. In order to calculate the half maximum effective concentration (EC50) that protects cells from death caused by ZIKV infection, MFI values from ZIKV MR766 control were subtracted from every condition and then these values were normalized over the mock control. These data were plotted and Hill 4 parameter sigmoidal regression was performed on Sigma Plot v.12.0 (Systat Software Inc., San Jose, CA, USA). 

### 2.8. Drug Cytotoxicity Assay

Vero cells, hBMEC, and NSC were incubated with medium containing chloroquine (1.56 to 200 μM) for 3 days (NSC) or 5 days (Vero and hBMEC). CellTiter Blue reagent (Promega) was added in each well, incubated for 12–16 h, and fluorescence was measured (560/590 nm). In order to calculate the 50% cytotoxicity concentration (CC50), MFI values were normalized over the untreated control. These data were plotted and Hill 4 parameter sigmoidal regression was performed on Sigma Plot v.12.0 (Systat Software Inc.). 

### 2.9. Time-of-Addition Assay

Vero cells were inoculated with ZIKV MR 766 at an MOI of 10 for 1 h at 4 °C. Cells were washed three times with cold PBS to remove unbound virus and treated with 50 μM chloroquine that was added at different time points: 0, 0.5, 3, 12, and 24 h post-infection. Conditioned media were collected at 30 h post-infection to analyze the production of virus particles through viral RNA content or the amount of infectious virus particles. Viral RNA was extracted and quantitative reverse transcription polymerase chain reaction (RT-qPCR) was performed. The titer of infectious virus particles was determined by plaque assay.

### 2.10. ZIKV Inhibition Assessed by Virus Production

Vero cells were infected with ZIKV MR766 or ZIKV BR strain at an MOI of 2 for 1 h at 4 °C. Virus input was washed three times with cold PBS and cells were treated with chloroquine (6.25 to 50 μM) for 48 h, and then the supernatant was collected and the RNA was extracted and analyzed by relative quantification by RT-qPCR. The supernatant was also evaluated by plaque assay to quantitate the infectious virus particles. 

### 2.11. Viral RNA Extraction and RT-qPCR

Viral RNA was extracted from 200 μL supernatant of infected cells using QIAmp MiniElute Virus Spin (QIAgen, Hilden, Düsseldorf ,DE), following the manufacturer’s recommendations. ZIKV detection was performed using One Step Taqman RT-qPCR (Thermo Fisher Scientific) on a 7500 Real-Time PCR System (Applied Biosystems) with primers and the probe described elsewhere [2]. Threshold cycle (CT) was determined and ∆CT (CT chloroquine treated − CT untreated) was calculated. The fold reduction of virus particles’ release, including defective viral particles, were calculated by 2^∆*C*t^.

### 2.12. Statistical Analysis

Mean and standard deviation (SD) were calculated for each assay. One way analysis of variance (ANOVA) was conducted using the non-parametric test (Kruskal–Wallis) followed by Dunn’s multiple comparisons test. A *p*-value of <0.05 was considered significant. All analyses were performed on GraphPad Prism v.7 (GraphPad Software, San Diego, CA, USA). The sample size is provided in the respective figure legends.

## 3. Results

### 3.1. Chloroquine Inhibits ZIKV Infection in Vero Cells 

**Figure 1:**
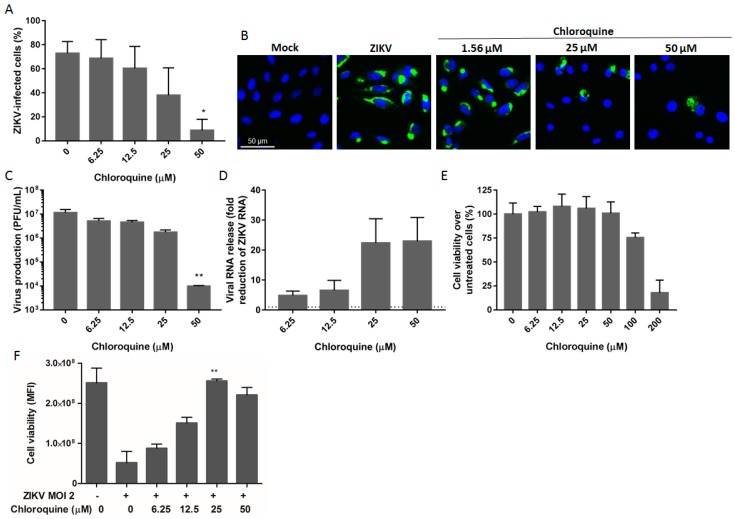
Inhibition of Zika virus (ZIKV) infection by chloroquine in Vero cells. (**A**) Vero cells were infected with ZIKV MR766 at a multiplicity of infection (MOI) of 2, treated with chloroquine for 5 days, and were then stained for the viral envelope protein and analyzed by flow cytometry; (**B**) Vero cells were infected, treated for 2 days, and ZIKV infection was evaluated by immunofluorescence staining with 4G2 antibody (green) and DAPI (blue); Infectious (**C**) or total (**D**) virus particles were quantified on the supernatant 48 h post-infection. Titers are expressed as plaque forming units (PFU) per milliliter. The dashed line represents no reduction in RNA levels; (**E**) Cell viability of uninfected cells treated with increasing concentrations of chloroquine was evaluated by fluorescence measurement at 560/590 nm after viability dye incubation; (**F**) Protection against ZIKV infection was evaluated through cell viability in ZIKV-infected Vero cells treated with chloroquine for 5 days. Data are represented as mean fluorescent intensity (MFI) ± standard deviation (SD) from two to four independent experiments. Statistical analysis was performed with the Kruskal–Wallis test and multiple comparisons with infected and untreated control corrected by Dunn’s test (* *p* < 0.05; ** *p* < 0,005).

We characterized the antiviral properties of chloroquine in Vero cells, a model widely used to study viral infections. Vero cells were infected with ZIKV MR766 at an MOI of 2 (i.e., 2 PFU/cell) and were then treated for 5 days with chloroquine in concentrations ranging from 6.25 to 50 μM. Viral infectivity was assessed using the 4G2 antibody, which detects flavivirus envelope E protein. We observed that chloroquine treatment decreased the number of ZIKV-infected cells in a dose-dependent manner. Flow cytometry analysis showed a reduction of 35% and 65% in ZIKV-infected cells when cultures were treated with 25 μM and 50 μM chloroquine, respectively, compared to untreated infected cells (Figure 1A). Immunofluorescence staining corroborated these results (Figure 1B) and additionally, chloroquine decreased the production of infectious (Figure 1C) and total (Figure 1D) virus particles, including defective viral particles, by ZIKV-infected cells. To confirm that viral inhibition is independent of chloroquine cytotoxicity, the viability of uninfected cells treated with chloroquine (1.56 to 200 μM) for 5 days was analyzed. Chloroquine did not impact cell viability at concentrations of 50 μM or lower (Figure 1E). We further analyzed whether chloroquine treatment could protect Vero cells from ZIKV infection as assessed by cell viability. Chloroquine, ranging from 12.5 to 50 μM, increased cell viability from 55% up to 100% (Figure 1F).

### 3.2. Chloroquine Inhibits Infection of Asian ZIKV Strains

**Figure 2:**
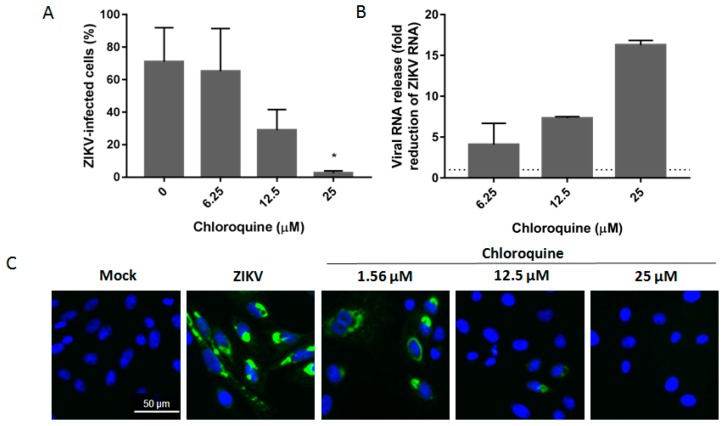
Chloroquine inhibits infection by Asian lineage. (**A**) Vero cells were infected with Brazilian ZIKV strain at an MOI of 2, treated with chloroquine at the indicated concentrations for 5 days, and the frequency of infected cells was evaluated by flow cytometry; (**B**) Vero cells were infected with Brazilian strain at an MOI of 2 and exposed to chloroquine for 48 h. The supernatant was collected and viral RNA was relatively quantified over the untreated infected control (**B**) or infectivity was analyzed by immunofluorescence with 4G2 antibody (**C**). The dashed line represents fold reduction on virus production of 1. Data are represented as mean ± SD from two independent experiments. Statistical significance was assessed by Kruskal–Wallis test and multiple comparisons with infected and untreated control corrected by Dunn’s test (* *p* < 0.05).

Microcephaly cases and neurological disorders have only been associated with infection with strains of ZIKV from the Asian lineage, detected in French Polynesia and in the Americas [6,9,29]. To determine the inhibition spectrum of chloroquine against ZIKV infection, Vero cells were infected with the Brazilian isolate of the Asian lineage (ZIKV BR). Chloroquine decreases the percentage of cells infected with the Brazilian isolate from 70% to 30% and 5% at 12.5 and 25 μM, respectively (Figure 2A,C). Moreover, viral RNA levels in the supernatant of Vero cells were quantified as a direct measurement of ZIKV infection. Treatment with 25 μM chloroquine led to a 16-fold reduction in the level of viral RNA detected in the supernatant (Figure 2B).

### 3.3. Chloroquine Inhibits Early Stages of ZIKV Infection 

**Figure 3:**
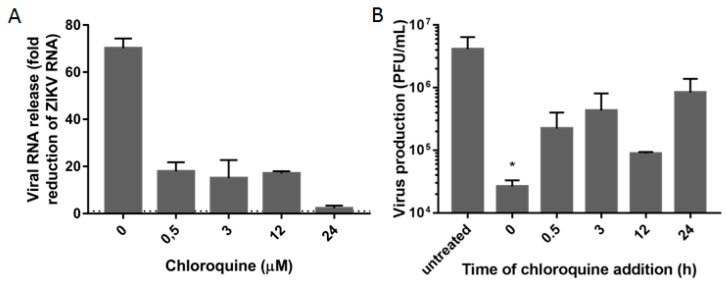
Early stages of infection are inhibited by chloroquine. Vero cells were infected with ZIKV MR766 at an MOI of 10 and 50 μM chloroquine was added at different times post-infection. At 30 h post-infection, the supernatant was collected and viral RNA (**A**) or infectious particles (**B**) were quantified. Viral RNA reduction is represented in fold change (2^∆*C*t^). The dashed line represents no reduction in RNA levels. Infectious particles are depicted as PFU/mL. Data are represented as mean ± SD from two independent experiments. Statistical analysis was performed with Kruskal–Wallis test and multiple comparisons with infected and untreated control corrected by Dunn’s test (* *p* < 0.05).

Inhibition of viral infection mediated by chloroquine can occur in both the early and later stages of the viral replication cycle [30,31]. To evaluate which step of the viral cycle was susceptible to inhibition, chloroquine was added to Vero cells at different time points post-infection with ZIKV MR766. The supernatant was collected 30 h post-infection and virus production was evaluated by relative quantification of viral RNA over the untreated control by RT-qPCR. Virus titers were also determined by plaque assay in Vero cells. Incubation of Vero cells with chloroquine at 0 h post-infection had a greater impact on the production of ZIKV particles, decreasing viral RNA 64-fold over the controls (Figure 3A). The addition of chloroquine from 30 min to 12 h post-infection was able to reduce virus release 9–20 fold over untreated, infected-cells. However, chloroquine added at 24 h post-infection had only a minor effect on viral production (Figure 3A). These results were confirmed by quantification of ZIKV infectious particles released after chloroquine treatment (Figure 3B). These data confirm that chloroquine interferes with the early stages of the ZIKV replication cycle.

### 3.4. Chloroquine Reduces ZIKV Infection in hBMEC, an In Vitro Model of the Blood–Brain Barrier 

**Figure 4:**
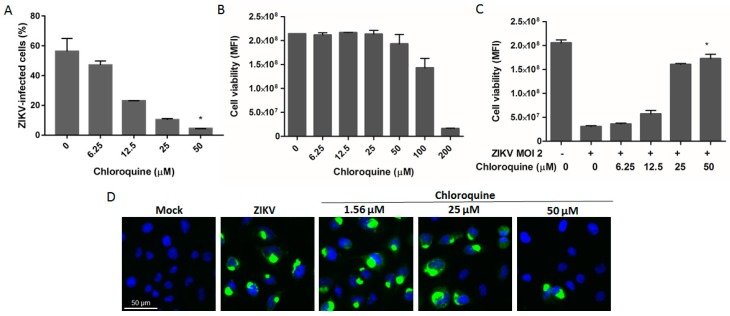
Chloroquine reduces the number of ZIKV-infected human brain microvascular endothelial cells (hBMECs). (**A**) hBMECs were infected with ZIKV MR 766 at an MOI of 2 followed by chloroquine treatment for 5 days. Cells were stained with the 4G2 antibody and analyzed by flow cytometry; (**B**) Uninfected hBMECs were incubated with chloroquine for 5 days and cell viability was analyzed; (**C**) Protection against ZIKV infection was measured through cell viability in chloroquine-treated ZIKV-infected cells; (**D**) Immunofluorescence with 4G2 antibody (green) and DAPI (blue) of ZIKV-infected cells treated with chloroquine for 5 days. Data are represented as mean ± SD from two independent experiments. Statistical significance was assessed by Kruskal–Wallis test and multiple comparisons with infected and untreated control corrected by Dunn’s test (* *p* < 0.05).

Considering that ZIKV infects hBMECs [32], we investigated whether chloroquine could inhibit viral infection of these cells. Chloroquine reduced 45% and 50% of the number of ZIKV MR766-infected hBMECs at 25 and 50 μM, respectively (Figure 4A,D). These concentrations are non-cytotoxic (Figure 4B), and protected approximately 80% of hBMECs from ZIKV infection as demonstrated by the increase in cell viability (Figure 4C).

### 3.5. Chloroquine Inhibits ZIKV Infection in Human Neural Progenitor Cells

**Figure 5:**
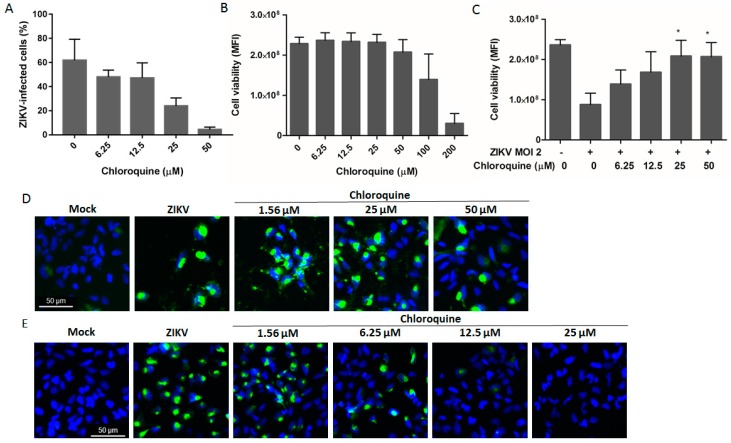
Chloroquine inhibits ZIKV infection in human neural stem cells (hNSCs). hNSCs were infected with ZIKV MR766 at an MOI of 2 and incubated with increasing concentrations of chloroquine for 4 days. (**A**) The frequency of ZIKV-infected cells was analyzed by 4G2 staining and flow cytometry; (**B**) Chloroquine cytotoxicity was assessed by the viability of uninfected hNSCs treated with chloroquine; (**C**) Chloroquine treatment protection from ZIKV infection was evaluated by cell viability measurement at 4 days post-infection. Immunofluorescence NSCs infected with ZIKV MR766 (**D**) or ZIKV BR (**E**) at an MOI of 2 and treated with chloroquine for 4 days with 4G2 antibody (green) and DAPI (blue). Data are represented as mean ± SD from two to three independent experiments. Statistical analysis was performed with Kruskal–Wallis test and multiple comparisons with infected and untreated control corrected by Dunn’s test (* *p* < 0.05).

Neural stem cells are key cells in the process of corticogenesis, giving rise to the three main cell types of the central nervous system: neurons, astrocytes, and oligodendrocytes. Depletion of the NSC pool is one of the main mechanisms responsible for primary microcephaly [33]. To evaluate if chloroquine could protect these cells from ZIKV MR766 infection, NSCs were exposed to up to 50 μM chloroquine for 4 days. Chloroquine treatment decreased the number of NSCs infected with ZIKV MR766 by 57%, and, through cell viability assessment protected 70% of NSCs from ZIKV infection, without cytotoxicity effects (Figure 5A–D). Similar results were observed when NSCs were infected with the Brazilian strain (Figure 5E).

### 3.6. Chloroquine Inhibits ZIKV Infection in Mouse Neurospheres 

**Figure 6:**
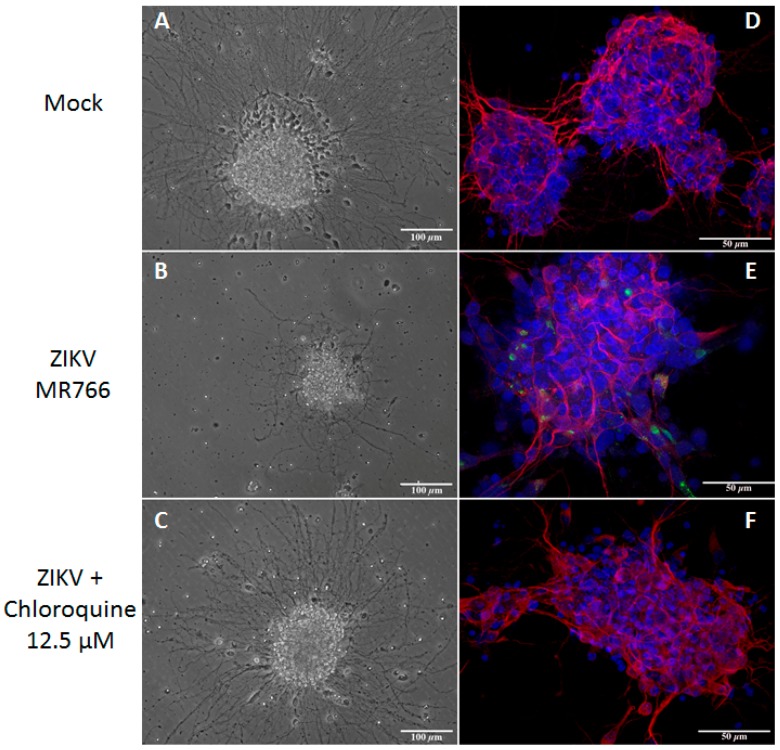
Chloroquine inhibits ZIKV infection in mouse neurospheres. Mouse neurospheres were infected with ZIKV MR766 (2.5 × 10^5^ PFU and were treated with chloroquine for 3 days. Neurospheres were analyzed by phase contrast microscopy (**A**–**C**), and triple stained for envelope viral protein (green), microtubule-associated protein 2 (Map-2, red), a neuron-specific protein, and DAPI (blue) (**D**–**F**).

Neuroprogenitor-enriched neurospheres, when subjected to differentiation culture conditions, can generate neurons. Our group recently demonstrated that ZIKV infection affected neurosphere size, neurite extension, and neuronal differentiation [34]. As we previously observed, neurospheres infected with ZIKV strain MR766 showed convoluted and misshapen neurites. Neurite extension was evaluated in chloroquine treated cultures by microtubule-associated protein 2 (Map2) staining and phase contrast microscopy and although many neurospheres were severely impacted by the infection, many others displayed the same general characteristics of mock-infected spheres indicating that chloroquine treatment rescued the neurite extension phenotype (Figure 6A–C). Furthermore, ZIKV infection decreased when neurospheres were treated with 12.5 μM chloroquine, as evaluated by 4G2 staining (Figure 6D–F).

## 4. Discussion

Chloroquine is known to be a non-specific antiviral agent, but its effect on the Zika virus replication has not been evaluated yet. This is the first report of inhibitory effects of chloroquine on ZIKV replication, which, given the ongoing epidemics, may become interesting both for the scientific knowledge of the virus and for the clinical perspective. 

Although Zika virus was first identified in Uganda in 1947, from January 2007 to April 2016, ZIKV transmission has been reported in 64 countries and territories [35]. The Zika virus disease is in general mild, but the recent positive correlation between infection, congenital malformations, and neurological damage in adults has intensified the need for therapeutic approaches. Prophylactic treatments for women intending to get pregnant in epidemic areas and travelers going to affected countries would represent relevant tools to reduce ZIKV transmission and avoid the spread of the disease by travelers. Moreover, a drug that blocks placental transfer of the virus could decrease the chance of vertical transmission in viremic pregnant women as was shown for HIV-infected pregnant women treated with antiretroviral therapy [36]. 

Here we demonstrated that chloroquine decreases the number of ZIKV-infected cells and protected cells from ZIKV infection as measured by cell viability at non-cytotoxic concentrations (Figure 1, Figure 4 and Figure 5). The EC50 or concentration of chloroquine that protected 50% of the cells from ZIKV infection assessed by cell viability, was 9.82–14.2 μM depending on the cell model and the CC50 ranged from 94.95 to 134.54 μM (Table 1). The values of EC50 obtained for ZIKV MR766 are lower than those obtained for DENV inhibition (~25 μM) and HIV inhibition (100 μM) [20,22]. Furthermore, we observed similar ZIKV inhibitory effects of chloroquine when tested on different ZIKV lineage infections (Figure 2 and Figure 5), supporting the idea that chloroquine could help to manage recent infections caused by Asian ZIKV lineage.

Although chloroquine has shown antiviral activity against a large spectrum of viruses in vitro, few clinical studies have been performed to evaluate chloroquine effects on patients with viral infections. Two clinical trial studies of chloroquine have been conducted to assess chloroquine treatment in patients infected with DENV [37,38]. One of the trials evaluated the benefits of chloroquine treatment for 3 days in patients infected with DENV and showed no reduction in the duration or intensity of DENV viremia or nonstructural 1 protein (NS1) antigenemia clearance [37]. However, a trend towards a reduction in the number of dengue hemorrhagic fever cases was noticed in the chloroquine-treated group [37]. A more recent clinical trial of chloroquine administration to DENV-infected patients, also for 3 days, showed that 60% of the patients in the chloroquine-treated group reported feeling less pain and showed improvement in the performance of daily chores during treatment [38]. Moreover, the symptoms returned after medication withdrawal. However, chloroquine treatment did not reduce the duration and intensity of the fever or duration of the disease [38]. The antiviral effect of chloroquine may be insufficient to produce a decrease in viral load or improvement of the disease progression when chloroquine/hydroxychloroquine is used in monotherapy. However, chloroquine may produce a significant antiflaviviral effect when used in combination therapies, as recently shown in a clinical trial of hydroxychloroquine plus ribavirin and interferon alpha in individuals infected with hepatitis C virus (HCV) [39]. In regard to the potential antiviral therapeutic combinations for Zika, a freshly published screening of drugs already approved for other clinical indications has resulted in the identification of more than 20 candidate drugs [40]. Of note, one of these is mefloquine, a compound related to chloroquine. In terms of safety for pregnant women, however, mefloquine is included in the B category, i.e., a drug for which the animal reproduction studies have failed to demonstrate a risk to the fetus and there are no adequate and well-controlled studies in pregnant women. Be that as it may, the aforementioned study corroborates our results using chloroquine, and provides new anti-ZIKV drugs that could be tested in combination with chloroquine.

Differing from mefloquine administration, the use of chloroquine during pregnancy was thoroughly evaluated and when prophylactic doses of chloroquine were administered for malaria (400 mg/week), no increment in birth defects was observed [41]. Higher concentrations of chloroquine (250 mg to 500 mg/day) were administered to pregnant women with severe diseases, such as lupus or rheumatoid arthritis. In these studies, few cases of abortion and fetal toxicity were observed. However, fetal toxicity or death could not be discarded as direct consequences of the disease itself. In addition, all term deliveries resulted in healthy newborns [19,42].

Chloroquine has been successfully tested in animal models. Twice daily administration of chloroquine (90 mg/kg) has been shown to increase the Balb/c mice survival rate to 80%–90% after infection with Ebola virus (EBOV) [43]. In a C57BL/6 mice model for coronavirus infection, chloroquine (50 mg/kg) protected 100% of 5-day-old suckling mice infected with human coronavirus OC43 (HCoV-OC43) when administered to pregnant mice 1 day prepartum [44]. A survival rate of 70% was observed in Balb/c mice infected with avian influenza H5N1 virus treated with chloroquine at 50 mg/kg/day [45]. These results suggest that chloroquine has the potential to inhibit ZIKV in in vivo mouse studies. 

Chloroquine is widely distributed to body tissues as well as its analogue hydroxychloroquine. The concentration of hydroxychloroquine in the brain is 4–30 times higher than in the plasma [46]. The concentration of chloroquine in the plasma reached 10 μM when a daily intake of 500 mg was prescribed to arthritis patients [47]. Chloroquine is able to cross the placental barrier and is supposed to reach similar concentrations in both maternal and fetal plasma [48]. Concentrations of chloroquine, similar to the EC50 values calculated here (Table 1), are achieved in the plasma in current chloroquine administration protocols and might reach the brain.

Different mechanisms for the chloroquine inhibition of viral infection have been described [49,50,51]. We observed a strong reduction in the release of ZIKV particles when the drug was added at 0 h post-infection (Figure 3), suggesting a higher impact on early stages of infection, possibly during fusion of the envelope protein to the endosome membrane. Chloroquine inhibits acidification of the endosome, consequently inhibiting the low pH-induced conformational changes required for the fusion of the envelope protein of flaviviruses with the endosomal membrane [52]. Chloroquine was also effective in decreasing virus release, although less pronouncedly and not statistically significant, when added after the early stages of virus infection (from 0.5 to 24 h post-infection), suggesting that later stages of the ZIKV replication cycle might also be affected (Figure 3). 

ZIKV was detected in the cerebrospinal fluid of ZIKV-infected adult patients that manifested meningoencephalitis, indicating that ZIKV invades the central nervous system through yet unknown mechanisms. Transcytosis through the endothelial cells of the blood brain barrier is a known mechanism of viral access to the central nervous system [53,54]. Here we demonstrated, by different methodologies, that chloroquine protects hBMEC, an immortalized cell line widely used as in vitro model of the blood–brain barrier [25], from ZIKV infection (Figure 4).

Recent studies showed that neural stem cells are highly permissive for ZIKV infection and one of the mechanisms proposed for the cause of microcephaly would be the depletion of the stem cell pool induced by ZIKV [10,11,55]. Our data showed that chloroquine inhibits the infection of human neural stem cells (Figure 5). Using the mouse neurospheres model to study neural stem cell differentiation into neurons, another process that might be disturbed in microcephaly, we observed that chloroquine inhibited the infection of neuronal progenitors and partially protected the ability of these cells to extend neurites (Figure 6). The protective effect of chloroquine on stem cells and committed progenitors is potentially a groundbreaking feature of this compound, as it would be prescribed to women at childbearing age that are traveling to affected countries and women planning pregnancy in endemic areas. This would decrease the chances of infection and thus fetal damage, especially to the developing brain.

Our results suggest that the chloroquine concentrations inhibiting ZIKV replication in vitro may overlap the highest drug concentrations detected in humans [56]. We therefore suggest that the therapeutic potential of chloroquine for Zika be subjected to further study.

## Figures and Tables

**Table 1:** Pharmacological parameters of chloroquine in each cell type against ZIKV MR766.

Cell Type	CC50	EC50	TI
Vero	134.54 ± 16.76 μM	9.82 ± 2.79 μM	13.70
hBMEC	116.61 ± 9.70 μM	14.20 ± 0.18 μM	8.21
hNSC	94.95 ± 9.38 μM	12.36 ± 2.76 μM	7.68

CC50: 50% cytotoxicity concentration; EC50: half maximal effective concentration; TI: therapeutic index (CC50/EC50). Data are represented as mean ± SD.

## References

[1] Dick G.W.A., Kitchen S.F., Haddow A.J. (1952). Zika virus. I. Isolations and serological specificity. Trans. R. Soc. Trop. Med. Hyg..

[2] Lanciotti R.S., Kosoy O.L., Laven J.J., Velez J.O., Lambert A.J., Johnson A.J., Stanfield S.M., Duffy M.R. (2008). Genetic and serologic properties of Zika virus associated with an epidemic, Yap State, Micronesia, 2007. Emerg. Infect. Dis..

[3] Faye O., Freire C.C.M., Iamarino A., Faye O., de Oliveira J.V.C., Diallo M., Zanotto P.M.A., Sall A.A. (2014). Molecular evolution of Zika virus during its emergence in the 20th century. PLoS Negl. Trop. Dis..

[4] Brasil P., Pereira J.P., Raja Gabaglia C., Damasceno L., Wakimoto M., Ribeiro Nogueira R.M., Carvalho de Sequeira P., Machado Siqueira A., Abreu de Carvalho L.M., Cotrim da Cunha D. (2016). Zika Virus Infection in Pregnant Women in Rio de Janeiro—Preliminary Report. N. Engl. J. Med..

[5] Martines R.B., Bhatnagar J., Keating M.K., Silva-Flannery L., Muehlenbachs A., Gary J., Goldsmith C., Hale G., Ritter J., Rollin D. (2016). Notes from the Field: Evidence of Zika Virus Infection in Brain and Placental Tissues from Two Congenitally Infected Newborns and Two Fetal Losses —Brazil, 2015. MMWR Morb. Mortal. Wkly. Rep..

[6] Mlakar J., Korva M., Tul N., Popović M., Poljšak-Prijatelj M., Mraz J., Kolenc M., Resman Rus K., Vesnaver Vipotnik T., Fabjan Vodušek V. (2016). Zika Virus Associated with Microcephaly. N. Engl. J. Med..

[7] Oliveira Melo A.S., Malinger G., Ximenes R., Szejnfeld P.O., Alves Sampaio S., Bispo De Filippis A.M. (2016). Zika virus intrauterine infection causes fetal brain abnormality and microcephaly: Tip of the iceberg?. Ultrasound Obstet. Gynecol..

[8] Melo A.S., Aguiar R.S., Amorim M.M., Arruda M.B., Melo F.O., Ribeiro S.T., Batista A.G., Ferreira T., Dos Santos M.P., Sampaio V.V. (2016). Congenital Zika Virus Infection: Beyond Neonatal Microcephaly. JAMA Neurol..

[9] Calvet G., Aguiar R.S., Melo A.S.O., Sampaio S.A., de Filippis I., Fabri A., Araujo E.S.M., de Sequeira P.C., de Mendonça M.C.L., de Oliveira L. (2016). Detection and sequencing of Zika virus from amniotic fluid of fetuses with microcephaly in Brazil: A case study. Lancet Infect. Dis..

[10] Garcez P.P., Loiola E.C., Madeiro da Costa R., Higa L.M., Trindade P., Delvecchio R., Nascimento J.M., Brindeiro R., Tanuri A., Rehen S.K. (2016). Zika virus impairs growth in human neurospheres and brain organoids. Science.

[11] Tang H., Hammack C., Ogden S.C., Jin P. (2016). Zika Virus Infects Human Cortical Neural Progenitors and Attenuates Their Growth. Stem Cell.

[12] Duffy M.R., Chen T.-H., Hancock W.T., Powers A.M., Kool J.L., Lanciotti R.S., Pretrick M., Marfel M., Holzbauer S., Dubray C. (2009). Zika virus outbreak on Yap Island, Federated States of Micronesia. N. Engl. J. Med..

[13] Bearcroft W.G. (1956). Zika virus infection experimentally induced in a human volunteer. Trans. R. Soc. Trop. Med. Hyg..

[14] Cao-Lormeau V.-M., Blake A., Mons S., Lastère S., Roche C., Vanhomwegen J., Dub T., Baudouin L., Teissier A., Larre P. (2016). Guillain–Barré Syndrome outbreak associated with Zika virus infection in French Polynesia: A case-control study. Lancet (Lond. Engl.).

[15] Carteaux G., Maquart M., Bedet A., Contou D., Brugières P., Fourati S., Cleret de Langavant L., de Broucker T., Brun-Buisson C., Leparc-Goffart I. (2016). Zika Virus Associated with Meningoencephalitis. N. Engl. J. Med..

[16] D’Ortenzio E., Matheron S., de Lamballerie X., Hubert B., Piorkowski G., Maquart M., Descamps D., Damond F., Yazdanpanah Y., Leparc-Goffart I. (2016). Evidence of Sexual Transmission of Zika Virus. N. Engl. J. Med..

[17] Deckard D.T., Chung W.M., Brooks J.T., Smith J.C., Woldai S., Hennessey M., Kwit N., Mead P. (2016). Male-to-Male Sexual Transmission of Zika Virus - Texas, January 2016. MMWR Morb. Mortal. Wkly. Rep..

[18] Browning D. (2014). Pharmacology of Chloroquine and Hydroxychloroquine. Hydroxychloroquine and Chloroquine Retinopathy.

[19] Levy M., Buskila D., Gladman D., Urowitz M., Koren G. (1991). Pregnancy Outcome Following First Trimester Exposure to Chloroquine. Am. J. Perinatol..

[20] Tsai W.P., Nara P.L., Kung H.F., Oroszlan S. (1990). Inhibition of human immunodeficiency virus infectivity by chloroquine. AIDS Res. Hum. Retrovir..

[21] Ooi E.E., Chew J.S.W., Loh J.P., Chua R.C.S. (2006). In vitro inhibition of human influenza A virus replication by chloroquine. Virol. J..

[22] Farias K.J.S., Machado P.R.L., da Fonseca B.A.L. (2013). Chloroquine inhibits dengue virus type 2 replication in Vero cells but not in C6/36 cells. Sci. World J..

[23] Zhu Y., Xu Q., Wu D., Ren H., Zhao P., Lao W., Wang Y., Tao Q., Qian X., Wei Y.-H. (2012). Japanese encephalitis virus enters rat neuroblastoma cells via a pH-dependent, dynamin and caveola-mediated endocytosis pathway. J. Virol..

[24] Boonyasuppayakorn S., Reichert E.D., Manzano M., Nagarajan K., Padmanabhan R. (2014). Amodiaquine, an antimalarial drug, inhibits dengue virus type 2 replication and infectivity. Antivir. Res..

[25] Stins M.F., Badger J., Sik Kim K. (2001). Bacterial invasion and transcytosis in transfected human brain microvascular endothelial cells. Microb. Pathog..

[26] Paulsen B.d.S., Maciel R.d.M., Galina A., da Silveira M.S., Souza C.d.S., Drummond H., Pozzatto E.N., Junior H.S., Chicaybam L., Massuda R. (2012). Altered Oxygen Metabolism Associated to Neurogenesis of Induced Pluripotent Stem Cells Derived From a Schizophrenic Patient. Cell Transplant..

[27] Yan Y., Shin S., Jha B.S., Liu Q., Sheng J., Li F., Zhan M., Davis J., Bharti K., Zeng X. (2013). Efficient and rapid derivation of primitive neural stem cells and generation of brain subtype neurons from human pluripotent stem cells. Stem Cells Transl. Med..

[28] Donald C.L., Brennan B., Cumberworth S.L., Rezelj V.V., Clark J.J., Cordeiro M.T., Freitas de Oliveira França R., Pena L.J., Wilkie G.S., Da Silva Filipe A. (2016). Full Genome Sequence and sfRNA Interferon Antagonist Activity of Zika Virus from Recife, Brazil. PLoS Negl. Trop. Dis..

[29] Cao-Lormeau V.-M., Roche C., Teissier A., Robin E., Berry A.-L., Mallet H.-P., Sall A.A., Musso D. (2014). Zika virus, French polynesia, South pacific, 2013. Emerg. Infect. Dis..

[30] Diaz-Griffero F., Hoschander S.A., Brojatsch J. (2002). Endocytosis is a critical step in entry of subgroup B avian leukosis viruses. J. Virol..

[31] Harley C.A., Dasgupta A., Wilson D.W. (2001). Characterization of herpes simplex virus-containing organelles by subcellular fractionation: role for organelle acidification in assembly of infectious particles. J. Virol..

[32] Bayer A., Lennemann N.J., Ouyang Y., Bramley J.C., Morosky S., Marques E.T.D.A., Cherry S., Sadovsky Y., Coyne C.B. (2016). Type III Interferons Produced by Human Placental Trophoblasts Confer Protection against Zika Virus Infection. Cell Host Microbe.

[33] Gilmore E.C., Walsh C.A. (2013). Genetic causes of microcephaly and lessons for neuronal development. Wiley Interdiscip. Rev. Dev. Biol..

[34] Campanati L., Higa L.M., Delvecchio R., Pezzuto P., De Filippis A.M.B., Aguiar R.S., Tanuri A. (2016). The Impact of African and Brazilian ZIKV isolates on neuroprogenitors. BioRxiv.

[35] WHO (2016). Zika Virus Microcephaly and Guillain-Barré Syndrome.

[36] Connor E.M., Sperling R.S., Gelber R., Kiselev P., Scott G., O’Sullivan M.J., VanDyke R., Bey M., Shearer W., Jacobson R.L. (1994). Reduction of maternal-infant transmission of human immunodeficiency virus type 1 with zidovudine treatment. Pediatric AIDS Clinical Trials Group Protocol 076 Study Group. N. Engl. J. Med..

[37] Tricou V., Minh N.N., Van T.P., Lee S.J., Farrar J., Wills B., Tran H.T., Simmons C.P. (2010). A randomized controlled trial of chloroquine for the treatment of dengue in vietnamese adults. PLoS Negl. Trop. Dis..

[38] Borges M.C., Castro L.A., da Fonseca B.A.L. (2013). Chloroquine use improves dengue-related symptoms. Mem. Inst. Oswaldo Cruz.

[39] Helal G.K., Gad M.A., Abd-Ellah M.F., Eid M.S. (2016). Hydroxychloroquine augments early virological response to pegylated interferon plus ribavirin in genotype-4 chronic hepatitis C patients. J. Med. Virol..

[40] Barrows N.J., Campos R.K., Powell S.T., Prasanth K.R., Schott-Lerner G., Soto-Acosta R., Galarza-Muñoz G., McGrath E.L., Urrabaz-Garza R., Gao J. (2016). A Screen of FDA-Approved Drugs for Inhibitors of Zika Virus Infection. Cell Host Microbe.

[41] Wolfe M.S., Cordero J.F. (1985). Safety of chloroquine in chemosuppression of malaria during pregnancy. Br. Med. J. (Clin. Res. Ed.).

[42] Parke A.L. (1988). Antimalarial drugs, systemic lupus erythematosus and pregnancy. J. Rheumatol..

[43] Madrid P.B., Chopra S., Manger I.D., Gilfillan L., Keepers T.R., Shurtleff A.C., Green C.E., Iyer L.V., Dilks H.H., Davey R.A. (2013). A Systematic Screen of FDA-Approved Drugs for Inhibitors of Biological Threat Agents. PLoS ONE.

[44] Keyaerts E., Li S., Vijgen L., Rysman E., Verbeeck J., Van Ranst M., Maes P. (2009). Antiviral activity of chloroquine against human coronavirus OC43 infection in newborn mice. Antimicrob. Agents Chemother..

[45] Yan Y., Zou Z., Sun Y., Li X., Xu K.-F., Wei Y., Jin N., Jiang C. (2013). Anti-malaria drug chloroquine is highly effective in treating avian influenza A H5N1 virus infection in an animal model. Cell Res..

[46] Titus E. (1989). Recent developments in the understanding of the pharmacokinetics and mechanism of action of chloroquine. Ther. Drug Monit..

[47] Mackenzie A.H. (1983). Dose refinements in long-term therapy of rheumatoid arthritis with antimalarials. Am. J. Med..

[48] Law I., Ilett K.F., Hackett L.P., Page-Sharp M., Baiwog F., Gomorrai S., Mueller I., Karunajeewa H.A., Davis T.M.E. (2008). Transfer of chloroquine and desethylchloroquine across the placenta and into milk in Melanesian mothers. Br. J. Clin. Pharmacol..

[49] Gonzalez-Dunia D., Cubitt B., de la Torre J.C. (1998). Mechanism of Borna disease virus entry into cells. J. Virol..

[50] Ferreira D.F., Santo M.P., Rebello M.A., Rebello M.C. (2000). Weak bases affect late stages of Mayaro virus replication cycle in vertebrate cells. J. Med. Microbiol..

[51] Savarino A., Boelaert J.R., Cassone A., Majori G., Cauda R. (2003). Effects of chloroquine on viral infections: An old drug against today’s diseases?. Lancet.

[52] Smit J.M., Moesker B., Rodenhuis-Zybert I., Wilschut J. (2011). Flavivirus cell entry and membrane fusion. Viruses.

[53] Dohgu S., Ryerse J.S., Robinson S.M., Banks W.A. (2012). Human immunodeficiency virus-1 uses the mannose-6-phosphate receptor to cross the blood-brain barrier. PLoS ONE.

[54] Suen W.W., Prow N.A., Hall R.A., Bielefeldt-Ohmann H. (2014). Mechanism of west nile virus neuroinvasion: A critical appraisal. Viruses.

[55] Qian X., Nguyen H.N., Song M.M., Hadiono C., Ogden S.C., Hammack C., Yao B., Hamersky G.R., Jacob F., Zhong C. (2016). Brain-Region-Specific Organoids Using Mini-bioreactors for Modeling ZIKV Exposure. Cell.

[56] Savarino A., Shytaj I.L. (2015). Chloroquine and beyond: exploring anti-rheumatic drugs to reduce immune hyperactivation in HIV/AIDS. Retrovirology.

